# Synthesis of Porous Organic Polymers with Tunable Amine Loadings for CO_2_ Capture: Balanced Physisorption and Chemisorption

**DOI:** 10.3390/nano9071020

**Published:** 2019-07-17

**Authors:** Xueying Kong, Shangsiying Li, Maria Strømme, Chao Xu

**Affiliations:** 1 Key Laboratory of Flexible Electronics (KLOFE), Institute of Advanced Materials (IAM), Nanjing Tech University (Nanjing Tech), 30 South Puzhu Road, Nanjing 211800, China; 2 Division of Nanotechnology and Functional Materials, Department of Engineering Sciences, Uppsala University, SE-75121 Uppsala, Sweden

**Keywords:** porous organic polymers, amine modification, CO_2_ separation, adsorption mechanism, chemisorption of CO_2_

## Abstract

The cross-coupling reaction of 1,3,5-triethynylbenzene with terephthaloyl chloride gives a novel ynone-linked porous organic polymer. Tethering alkyl amine species on the polymer induces chemisorption of CO_2_ as revealed by the studies of ex situ infrared spectroscopy. By tuning the amine loading content on the polymer, relatively high CO_2_ adsorption capacities, high CO_2_-over-N_2_ selectivity, and moderate isosteric heat (*Q*_st_) of adsorption of CO_2_ can be achieved. Such amine-modified polymers with balanced physisorption and chemisorption of CO_2_ are ideal sorbents for post-combustion capture of CO_2_ offering both high separation and high energy efficiencies.

## 1. Introduction

The long-term increasing CO_2_ emission from combustion of fossil fuels is widely considered as the main reason for the global climate change and associated environmental issues [1]. Post-combustion carbon capture dealing with separation of CO_2_ from flue gases is a feasible approach to reduce industrial CO_2_ emissions and to gain control over the atmospheric CO_2_ concentration, and the method has the advantage of allowing quite simple retrofit design of required instrumentation into existing power plants [2]. However, the main challenge for the post-combustion technology is that the low concentration of CO_2_ (ca. 5%−15 v%) in flue gases usually results in low separation efficiency [3]. Amine scrubbing, a mature technique using aqueous amine solution to absorb CO_2_ from mixed gases, has been applied in natural gas purification and CO_2_ capture for more than a half century [4] and the technique is currently employed to create large scale pilot facilities in, amongst others, Norway [5]. The strong chemical interactions between amine and CO_2_ molecules endow the high efficiency for CO_2_ capture and separation. However, the energy consumption for amine reactivation arising from heating the aqueous amine solution is high. In addition, the use of amine might cause amine leakage and serious corrosion to the equipment. Therefore, it is highly desirable to develop new materials for post-combustion carbon capture that can be operated in an economical and environmentally friendly manner.

Porous materials with high surface areas and high volumes of narrow pores are ideal solid sorbents for adsorption-driven CO_2_ capture, in which CO_2_ molecules can be selectively adsorbed onto the surface or captured in the narrow pores [6,7]. For example, traditional zeolites and activated carbons have been extensively studied for CO_2_ capture owing to their high microporosities and relatively high CO_2_ adsorption capacities [8,9,10,11,12,13,14]. However, the hydrophilicity of zeolites and the broad pore size distributions of activated carbons have significantly limited their performances in CO_2_ capture and separation. Emerging porous materials such as metal−organic frameworks (MOFs) [15,16,17] and porous organic polymers (POPs) [18,19,20,21,22,23] are of great interest for CO_2_ capture because of their large surface areas and tunable pore sizes. POPs constitute a type of porous materials created by linking pure organic monomers, usually aromatic or conjugated, via strong covalent bonds [24,25]. The diverse synthesis possibilities of POPs allows precise control of their nanoporous structure and surface chemistry at the molecular level, aiming to increase the CO_2_ adsorption capacity and selectivity of CO_2_ over other gases by thermodynamic effects [26,27,28,29,30,31]. Several studies reported on post-modification of POPs with alkyl amines for CO_2_ capture [32,33,34,35,36,37,38,39]. The strong CO_2_−amine interactions on the amine-modified POPs led to significantly enhanced CO_2_ adsorption capacity and increased CO_2_-over-N_2_ selectivity. However, the strong interactions, interpreted by the high isosteric heat (*Q*_st_) of adsorption (up to 80 kJ mol^−1^) [35,37], require high energy input to reactivate the sorbents in the process of temperature swing adsorption (TSA) or vacuum swing adsorption (VSA) [40]. In this context, it would be great of interest to balance the trade-off between the separation efficiency and energy efficiency. Here, we report a strategy to tune the amine density on a novel ynone-linked POP (y-POP) by the post-modification approach, which enables balancing of the effects of physisorption and chemisorption of CO_2_ and optimization of the CO_2_ adsorption capacity, CO_2_-over-N_2_ selectivity and heat of adsorption of CO_2_.

## 2. Results and Discussion

**Scheme 1:**
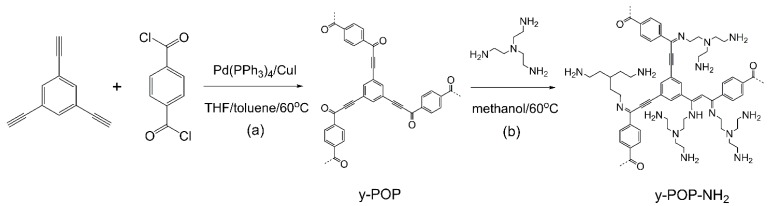
(**a**) Synthesis of ynone-linked porous organic polymer (y-POP) by the Sonogashira coupling reaction; (**b**) amine modification of y-POP.

POPs can be synthesized from various organic reactions, of which coupling reactions constitute the most commonly used routes [41]. For example, Zhu et al. reported on the Yamamoto homo-coupling reaction of tetrakis(4-bromophenyl)methane for the synthesis of a porous aromatic framework (PAF-1) [42]. The tetrahedral shaped monomer resulted in a three-dimensional framework of PAF-1 possessing an ultrahigh surface area of 5600 m^2^ g^−1^. Cooper and Jiang’s groups synthesized a number of conjugated microporous polymers (CMPs) by Suzuki, Sonogashira, and Glaser coupling reactions, which showed great potential in photocatalysis, light harvesting, etc. [43,44,45,46]. Recently, Son et al. developed a carbonylative Sonogashira coupling reaction of phenyl alkynes and phenyl halides in the presence of carbon monoxide, which gave a redox-active CMP that can be used as electrode material for electrochemical energy storage devices [47]. Therefore, the exploration of organic reactions to form novel structures in POPs could enrich their properties and applications. As we know, the cross-coupling reaction between terminal alkynes and acyl chloride under Sonogashira conditions forms conjugated *α*,*β*-alkynic ketones, also known as ynones [48]. More interestingly, further reaction of the ynones with alkyl amines could form imine compounds by two possible routes [48,49,50,51,52]. One is that of conjugate addition of the amine to *α*,*β*-alkynic ketone with formation of enaminone, which can be further converted into the imine compound via the nucleophilic addition in the presence of excess of amine. Another approach is direct nucleophilic addition of the amine to the ketone group forming the imine or enaminone compound. However, to the best of our knowledge, the direct coupling of terminal alkyne with acyl chloride has never been reported for the synthesis of POPs. In this context, we attempted the cross-coupling of 1,3,5-triethynylbenzene with terephthaloyl chloride under Sonogashira conditions for the synthesis of y-POP, which was catalyzed by bis(triphenylphosphine)palladium(II) dichloride and copper(I) iodide in the presence of trimethylamine as a base (Scheme 1a). The conjugated structure of the ynones and the aromatic monomers endow rigidity and stability of y-POP. In order to graft amine species onto the polymer, the as-synthesized y-POP was treated by tris(2-aminoethyl)amine (**tren**) in a methanol solution (Scheme 1b). The density of amine species tethered on the polymer was finely controlled by tuning the concentration of **tren** in the methanol solution. Specifically, treatment of y-POP in the methanol solution of **tren** (1, 5, 20 v%) yields the amine modified polymer y-POP-NH_2_, denoted as y-POP-A1, y-POP-A2, and y-POP-A3, respectively. Based on the thermogravimetric analyses (Figure S1), the amine loading content on the y-POP-NH_2_ can be roughly calculated to 12%, 16%, and 19% for y-POP-A1, y-POP-A2, and y-POP-A3, respectively.

**Figure 1:**
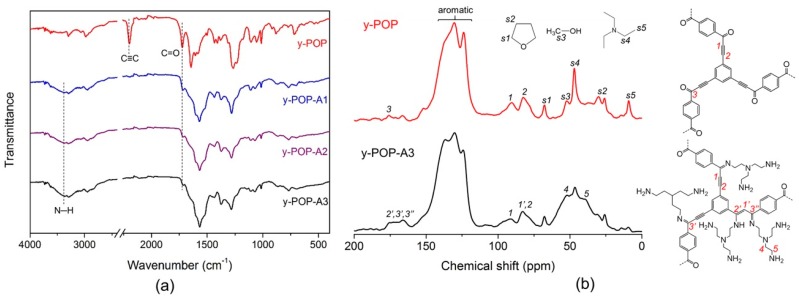
(**a**) Infrared (IR) and (**b**) ^13^C nuclear magnetic resonance (NMR) spectra of ynone-linked porous organic polymer (y-POP) and y-POP-NH_2_.

The molecular structures of y-POP and y-POP-NH_2_ were examined by both Fourier transform infrared (IR) and solid-state ^13^C nuclear magnetic resonance (NMR) spectroscopy. The IR spectrum of y-POP shows strong bands at 2200 and 1720 cm^−1^, corresponding to the stretching vibrations of alkyne (−C≡C−) and carbonyl (−C=O), respectively (Figure 1a). In general, the central −C≡C− group with a high degree of symmetry displays a very weak IR stretching band [45]; however, the intensity of the IR band can be significantly increased by introducing conjugated structures [47,53]. Therefore, the intense IR band observed for the alkyne group can be correlated to the formation of conjugated structure with a carbonyl group (−C≡C(=O)−). The study of the solid-state ^13^C NMR spectrum also confirms the formation of ynone species in the polymer. The bands at chemical shifts of 91 and 83 ppm can be assigned to carbon atoms (*1*, *2*) of alkyne bonds (Figure 1b and Figure S2). The characteristic band for carbon atoms (*3*) in carbonyl groups was observed at 176 ppm. Upon amine modification, the intensity of the IR band at 1720 cm^−1^ was gradually decreased with increasing amine loading, indicating that the carbonyl groups were consumed by the amine. In addition, the IR band intensity at 2200 cm^−1^ and NMR band intensity at 91 and 83 ppm were decreased in y-POP-NH_2_ compared to y-POP due to the transformation of the alkyne groups to enaminones. The broad shoulder bands at ~1660 cm^−1^ in the IR spectra of y-POP-NH_2_ can be assigned to the imine stretching vibrations. Consistently, the broad shoulder bands at the NMR chemical shift of 175−168 ppm for y-POP-NH_2_ indicate the formation of enamine and imine bonds [52,54]. The broad IR bands at 3370 cm^−1^ observed for y-POP-NH_2_ are assigned to the characteristic N−H stretching modes for the primary amine. The chemical shifts at 52 and 39 ppm for y-POP-NH_2_ correspond to the carbons in tethered **tren** species. The three major peaks at 137, 130 and 123 ppm are assigned to the aromatic carbons in y-POP and y-POP-NH_2_. Other peaks in the range of 60−10 ppm can be assigned to the carbon atoms in solvent molecules (tetrahydrofuran, methanol, triethylamine) adsorbed in the pores of y-POP. Collectively, the studies of the IR and ^13^C NMR spectra confirmed that ynone-linked POP was successfully synthesized and the **tren** molecules were chemically tethered on y-POP-NH_2_.

**Figure 2:**
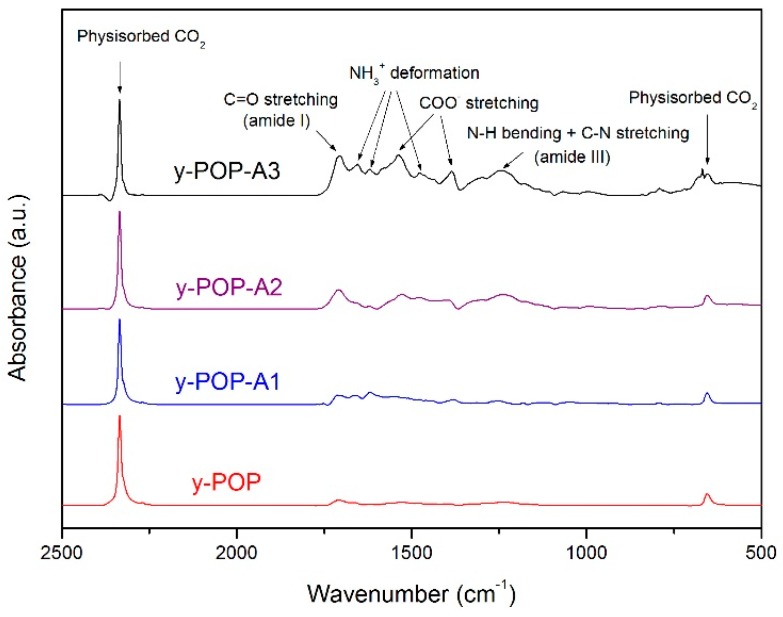
Infrared (IR) spectra of adsorbed CO_2_ on ynone-linked porous organic polymer (y-POP) and y-POP-NH_2_.

As a strong organic base with a pKb value ≈4, the tethered **tren** species on y-POP-NH_2_ could potentially attract CO_2_ molecules by the chemisorption effect. To investigate the CO_2_ adsorption mechanism, we designed an ex situ IR experiment to study the molecular interactions between CO_2_ and the polymers. The polymer sample was grinded with KBr and pressed into a transparent pellet, followed by drying at 100 °C for 12 h. The degassed pellet was used to record the IR background spectrum in a transmission model. The pellet was subsequently flashed with CO_2_ for 2 h at room temperature and thereafter a transmission IR spectrum was recorded again. The differences between the two spectra revealed the adsorbed CO_2_ on the polymers, as shown in Figure 2. The intense bands at frequencies of 2335 and 653 cm^−1^ in the spectra correspond to the physisorbed CO_2_ molecules, which can be assigned to the asymmetric stretching and deformation vibration of C=O, respectively [55]. Physisorption clearly dominates the CO_2_ adsorption on y-POP as no extra band was observed in the IR spectrum. In contrast, significant IR bands in the frequency region of 1750−1000 cm^−1^ were observed for y-POP-NH_2_, which indicates that the tethered amine species induced chemisorption of CO_2_. Obviously, a higher amine loading content in y-POP-NH_2_ resulted in a stronger IR intensity, suggesting the enhanced effect of chemisorption of CO_2_. The band at the frequency of 1704 cm^−1^ was assigned to C=O stretching (amide I), which is a characteristic indication of the formation of carbamic acid or carbamate species from the reaction between CO_2_ and the amine groups on y-POP-NH_2_ [56,57,58]. The broad band at 1243 cm^−1^ was assigned to a combination of N−H bending and C−N stretching (amide III) [59]. In addition, the bands at 1655, 1618, and 1475 cm^−1^ can be assigned to asymmetric deformation of NH_3_^+^ and the signals at 1538 and 1385 cm^−1^ can be associated to asymmetric stretching of COO^−^ [55,60,61,62,63]. Therefore, we could speculate that chemisorption of CO_2_ on y-POP-NH_2_ forms ammonium carbamate ion pairs (Scheme S1).

**Figure 3:**
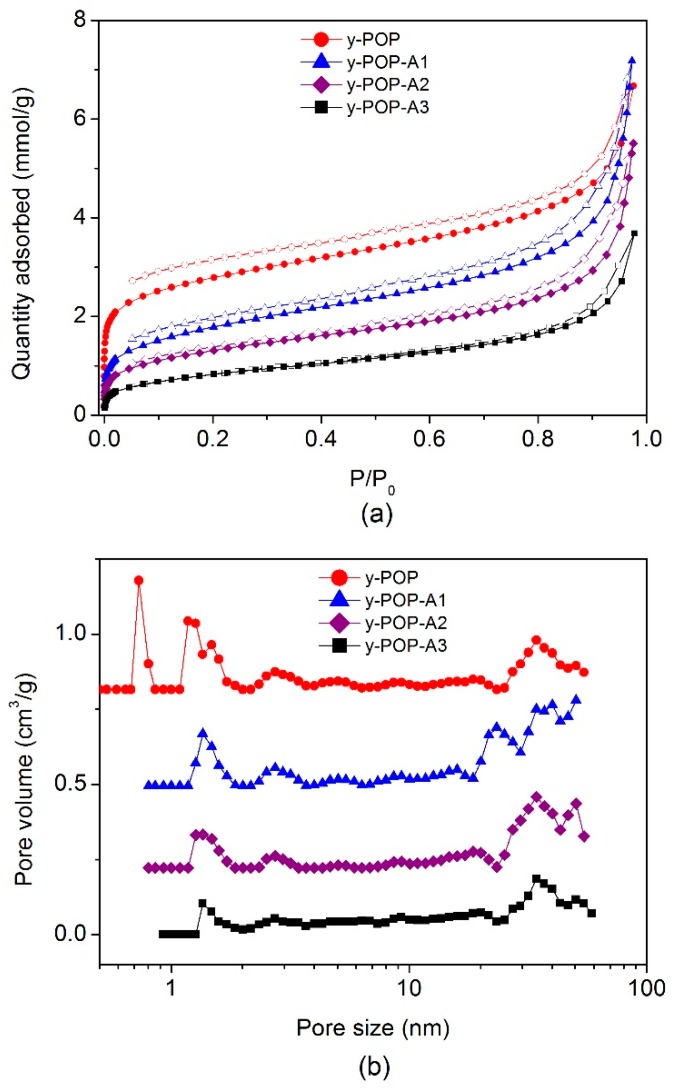
(**a**) N_2_ adsorption/desorption isotherms of ynone-linked porous organic polymer (y-POP) and y-POP-NH_2_ recorded at 77 K; (**b**) Pore size distribution analyses of y-POP and y-POP-NH_2_ based on the adsorption branches using the density functional theory model.

The porosities of the polymers were analyzed by N_2_ sorption measurements at 77 K, as illustrated in Figure 3a. The sorption isotherm of y-POP displays rapid N_2_ adsorption at low relative pressures (*p*/*p*_0_ < 0.05), which is characteristic for microporous materials. The significant N_2_ uptake at high relative pressures (*p*/*p*_0_ > 0.8) and the accompanied hysteresis loop between the adsorption and desorption branches suggest the presence of mesopores in y-POP arising from the inter-particle cavities observed in SEM images (Figure S3). Such mesopores would facilitate the mass transportation and increase the adsorption kinetics during the sorption processes. Pore size distribution analyses showed that y-POP had ultramicropores (pore size: 0.74 nm), micropores (pore size: 1.2 nm) and mesopores (pore size: 34 nm). As expected, amine modification on y-POP resulted in disappearance of the ultramicropores and decrease in both micropore volumes and total pore volumes due to the pore blocking effect of the **tren** species (Figure 3b). Consistently, the specific surface area of the y-POP-NH_2_ was gradually decreased with increasing amine loading: the specific surface area values being 226, 145, 107, and 84 m^2^ g^−1^, for of y-POP, y-POP-A1, y-POP-A2, and y-POP-A3, respectively.

**Figure 4:**
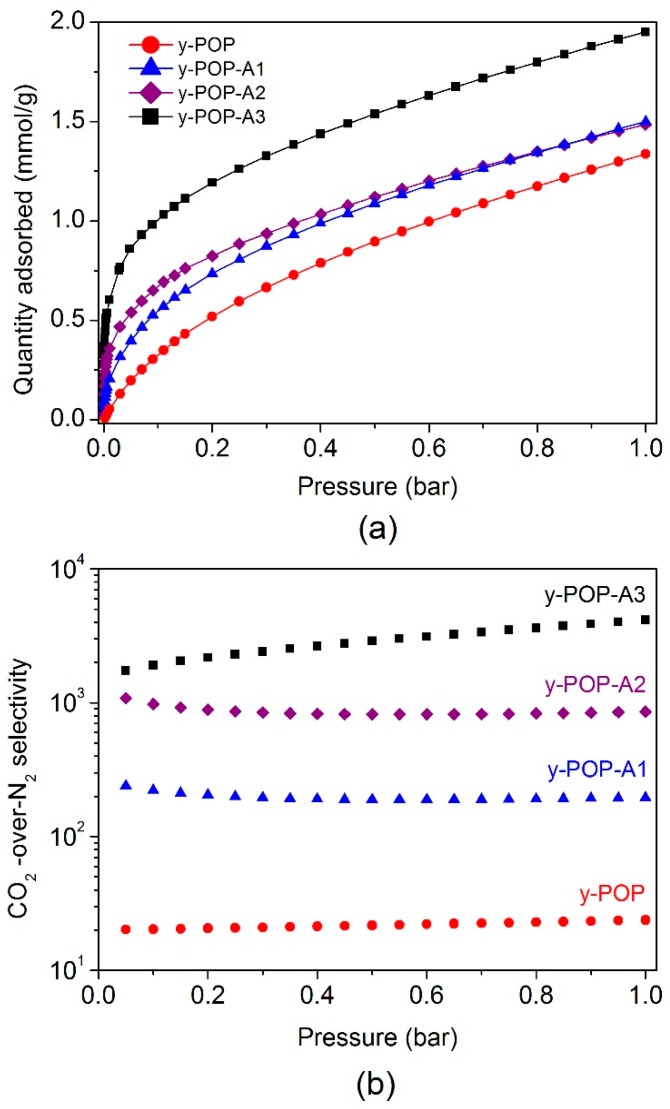
(**a**) CO_2_ adsorption isotherms of ynone-linked porous organic polymer (y-POP) and y-POP-NH_2_ with different amine loadings at 273 K; (**b**) CO_2_-over-N_2_ selectivity of y-POP and y-POP-NH_2_ calculated by ideal adsorbed solution theory.

Given the microporous structure and the strong chemisorption of CO_2_ induced by the high amount of amine species, we anticipated that y-POP-NH_2_ would have much higher CO_2_ adsorption capacities and higher CO_2_-over-N_2_ selectivity than the corresponding values of unmodified y-POP. Figure 4a compares the CO_2_ adsorption isotherms of y-POP and y-POP-NH_2_ with different amine densities at 273 K. Although the specific surface areas of the polymers were reduced after the amine modification, y-POP-NH_2_ had significantly higher CO_2_ adsorption capacities than the substrate polymer of y-POP. With the increase of amine loading, the CO_2_ adsorption capacity of y-POP-NH_2_ gradually increased up to 1.11 and 1.88 mmol g^−1^ at 0.15 and 1 bar (273 K), respectively, which were 158% and 40% higher than the corresponding values of y-POP (0.43 mmol g^−1^ at 0.15 bar; 1.34 mmol g^−1^ at 1 bar, 273 K). In addition, the sorbents can be easily reactivated and showed excellent adsorption recyclability. For example, y-POP-A1 retained 97% of its CO_2_ adsorption capacity after 5 cycles at 293 K (Figure S6). In order to evaluate the potential of the polymers for capturing CO_2_ from flue gases, we calculated the CO_2_-over-N_2_ selectivity from their single component adsorption data recorded at 273 K (Figure 4a and Figure S7) using ideal adsorption solution theory (IAST) [13,35,64,65]. The CO_2_ and N_2_ adsorption isotherms were fitted by dual-site and single-site Langmuir equation, respectively (Figure S8 and Table S1). The CO_2_-over-N_2_ selectivity is defined as S = (xCO_2_/yCO_2_)/(xN_2_/yN_2_), where x and y are the molar fractions of the gas in the adsorbed and bulk phases, respectively. A simulated gas mixture of 15 v% CO_2_/85 v% N_2_ was used for the calculation. As illustrated in Figure 4b, the substrate polymer y-POP showed a relatively low CO_2_-over-N_2_ selectivity of 20. In contrast, y-POP-NH_2_ containing amine species displayed much higher selectivity up to 4.15 × 10^3^. In addition, we have calculated Henry’s law initial slope selectivity for the polymers (Figure S9), which are comparable to the values obtained from the IAST calculations.

The binding affinity of the studied polymers toward CO_2_ was revealed by the *Q*_st_ values, which can be calculated from the temperature dependent CO_2_ adsorption isotherms (273, 283, and 293 K) using the Clausius–Clapeyron equation. The substrate polymer of y-POP had a relatively low *Q*_st_ value of 29.0 kJ mol^−1^, which is characteristic for the physisorption of CO_2_. As expected, y-POP-NH_2_ showed gradually increased *Q*_st_ values (y-POP-A1: 46.8 kJ mol^−1^; y-POP-A2: 62.2 kJ mol^−1^; y-POP-A3: 76.5 kJ mol^−1^) with increasing amine loading (Figure S5). The high *Q*_st_ values indicate the effect of chemisorption of CO_2_ on y-POP-NH_2_, which is consistence with the ex situ IR results.

Table 1 summarizes CO_2_ adsorption capacities, CO_2_-over-N_2_ selectivity, and *Q*_st_ values for the four studied polymers. It is immediately clear that amine modification on the polymer significantly increases the efficiency for CO_2_ capture and separation due to the effect of chemisorption of CO_2_. In addition, the efficiency was proportional to the amine loading density on the polymer. However, the polymers containing high amine loading contents had relatively high *Q*_st_ values, which means that reactivation of the sorbents requires a high energy consumption. Noteworthy, the sample y-POP-A1 had relatively high CO_2_ adsorption capacities (0.65 mmol g^−1^ at 0.15 bar; 1.50 mmol g^−1^; 273 K) and high CO_2_-over-N_2_ selectivity of 271 (273 K). The selectivity is comparable to some top-performing sorbents of amine-modified POPs [32], MOFs [66] and silica [67] that indicates the potential of using y-POP-A1 for efficient CO_2_ capture. In contrast, y-POP-A1 demonstrates a moderate *Q*_st_ value of 46.8 kJ mol^−1^ at a low coverage of CO_2_ (0.2 mmol g^−1^), which is much lower than those of amine-modified sorbents (mmen-CuBTTri: 96 kJ mol^−1^; [66] PP1-2-tren: 80 kJ mol^−1^; [37] NTU-1: 75 kJ mol^−1^; [35] PEI (40 wt%) ⊂ PAF-5: 68.7 kJ mol^−1^ [38]; PPN-6-CH_2_-TETA: 63 kJ mol^−1^ [32]). Therefore, the balanced effects of physisorption and chemisorption of CO_2_ on y-POP-A1 would offer both high separation efficiency and high energy efficiency for post-combustion capture of CO_2_ from flue gases.

## 3. Conclusions

To conclude, a novel ynone-linked POP was synthesized and its molecular structure was fully characterized by IR and ^13^C NMR spectroscopy. The polymer was further used as a substrate to tether alkyl amine species by post modification. The ex situ IR results revealed that the amine species on the polymers could induce chemisorption of CO_2_ with formation of ammonium carbamate ion pairs. As a result, the amine-modified polymers showed high CO_2_ adsorption capacities, high CO_2_-over-N_2_ selectivity, as well as high *Q*_st_ values. Remarkably, the amine density on the polymers can be finely controlled by a molecular engineering approach, which allows balancing the physisorption and chemisorption of CO_2_ to reach a high separation efficiency, excellent recyclability, high energy efficiency for CO_2_ capture and separation. The use of this strategy in the design of amine-modified porous solids (e.g., mesoporous silica, MOFs, clay, etc.) would offer highly efficient sorbents for post-combustion capture of CO_2_.

## Figures and Tables

**Table 1:** A summary of specific surface area (S_BET_), CO_2_ adsorption capacity (273 K), CO_2_-over-N_2_ selectivity (273 K), and *Q*_st_ of CO_2_ adsorption at the low coverage of CO_2_ for ynone-linked porous organic polymer (y-POP) and y-POP-NH_2_ with different amine densities.

Sample	Amine Loading	S_BET_ (m^2^ g^−1^)	CO_2_ Uptake (mmol g^−1^)		CO_2_/N_2_ Selectivity		*Q*_st_ (kJ mol^−1^)
			0.15 bar	1 bar	IAST	Henry’s Law	
y-POP	0	226	0.43	1.34	20	22	29.0
**y-POP-A1**	**12%**	**145**	**0.65**	**1.50**	**239**	**216**	**46.8**
y-POP-A2	16%	107	0.76	1.49	1083	750	62.2
y-POP-A3	19%	84	1.11	1.95	4154	3806	76.5

## Supplementary Materials

The following are available online at https://www.mdpi.com/2079-4991/9/7/1020/s1. Figure S1. Thermogravimetric analysis curves of y-POP and y-POP-NH2. Figure S2. Solid-state 13C NMR spectra of y-POP, y-POP-A1, y-POP-A2, and y-POP-A3. Figure S3. SEM images of y-POP, y-POP-A1, y-POP-A2, and y-POP-A3. Figure S4. Powder X-ray diffraction patterns of y-POP and y-POP-NH2 showing the polymers are mainly amorphous. Figure S5. CO_2_ adsorption isotherms of y-POP, y-POP-A1, y-POP-A2, and y-POP-A3 recorded at 293 K. Figure S6. CO_2_ adsorption-desorption cycles for y-POP-A1 recorded at 293 K. Figure S7. N_2_ adsorption isotherms of y-POP, y-POP-A1, y-POP-A2, and y-POP-A3 recorded at 273 K. Figure S8. CO_2_ (■) and N_2_ (▲) adsorption data and of y-POP and y-POP-NH2 recorded at 273 K. The red solid lines show the fitting results of the data: The CO_2_ and N_2_ adsorption data was fitted by a dual-site and single-site Langmuir model, respectively. Detail fitting results are given in Table S1. The fitted parameters from the single adsorption data were used to predict the IAST selectivity. Figure S9. The CO_2_ and N_2_ adsorption data of (a) y-POP, (b) y-POP-A1, (c) y-POP-A2, and (d) y-POP-A3 at low partial pressures at 273 K and the linearly fitted results. Henry’s law CO_2_-over-N_2_ selectivities were calculated from the initial slopes of the CO_2_ and N_2_ isotherms. Table S1. Fitting parameters for the CO_2_ and N_2_ adsorption data recorded at 273 K. Scheme S1. Possible mechanism of chemisorption of CO_2_ on y-POP-NH2 with high amine loadings.
