# Supplementary material for: Synthesis of Porous Organic Polymers with Tunable Amine Loadings for CO2 Capture: Balanced Physisorption and Chemisorption
Source: Nanomaterials (Basel). 2019 Jul 17;9(7):1020. doi: 10.3390/nano9071020 (PMC6669882; doi:10.3390/nano9071020)
Supplement: Supplementary file 1 [file nanomaterials-09-01020-s001.pdf]

Supporting Information

Synthesis of porous organic polymers with tunable amine loadings for CO_2 capture: balanced physisorption and chemisorption

Xueying Kong, [ab] *Shangsiying Li,* [a] *Maria Strømme,* [b] *Chao Xu**[ab]

[a] Key Laboratory of Flexible Electronics (KLOFE), Institute of Advanced Materials (IAM),

Nanjing Tech University (Nanjing Tech), 30 South Puzhu Road, Nanjing 211800,

[b] Division of Nanotechnology and Functional Materials, Department of Engineering

Sciences, Uppsala University, Uppsala SE-75121, Sweden. E-mail: chao.xu@angstrom.uu.se

Experiment sections

1. Materials

1,3,5-triethynylbenzene (> 95% purity), terephthaloyl chloride (> 99% purity), tris(2-aminoethy)amine (**tren**) (> 97% purity), bis(triphenylphosphine)palladium dichloride (> 99% purity), copper iodide (> 99% purity) were purchased from Alfa-Aesar. All solvents were purchased from Sigma-Aldrich. All chemicals were used without further purification.

2. Synthesis of y-POP

y-POP was synthesized under N_2 atmosphere. 1,3,5-triethynylbenzene (0.5 mmol, 79 mg), bis(triphenylphosphine)palladium dichloride (0.018 mmol, 12.6 mg) and copper iodide (0.06 mmol, 11.4 mg) were added to a 50 mL round-bottomed flask. A mixed solvent of 2.5 mL THF and 2.5 mL toluene was added to form a brown solution. A colorless solution of terephthaloyl chloride (0.75 mmol, 153 mg) dissolved in a mixed solvent of 2.5 mL THF and 2.5 mL was added to the above solution. Finally, 5 mL triethylamine was added and the mixture was heated at 60 ℃ for 24 h, which formed yellow precipitates. The solids were collected by filtration and then purified by Soxhlet extraction using THF and methanol for 12 h, respectively. The obtained product (y-POP) was dried at 60 ℃ for 24 h.

3. Synthesis of y-POP-NH_2

y-POP (100 mg) and **tren** (0.1 mL) was mixed in 9.9 mL methanol. The mixture was stirred at 60 ℃ for 24 h. The obtained yellow precipitates were collected by filtration and Soxhlet extracted by methanol. The product (y-POP-A1) was dried at 60 ℃ for 24 h. y-POP-A2 and y-POP-A3 were prepared by the same procedures instead of using 0.5 mL **tren**/9.5 mL methanol and 2 mL **tren**/8 mL methanol, respectively.

4. Characterization

The infrared (IR) spectra were recorded on a Bruker Tensor 27 spectrometer in the transmission model. The solid-state $^{13}C\{^1H\}$ NMR spectra were recorded on a Bruker AVWBIII600 spectrometer. Scanning electron microscope (SEM) images were collected on a FEG SEM instrument (Zeiss, Leo Gemini 1530). Thermogravimetric analysis (TGA) was performed on a thermogravimetric analyzer (Mettler Toledo, TGA/SDTA851e) under a N_2 flow (60 mL/min) between 25 and 800 ℃ with a heating rate of 5 ℃/min. Powder X-ray diffraction (XRD) patterns were recorded on a Bruker Focus D8 diffractometer with a Cu-Kα radiation (λ = 1.5418 Å). N_2 and CO_2 sorption isotherms were recorded on a Micromeritics ASAP 2020 surface area and pore size analyzer. The samples were degassed at 100 ℃ under a kinetic vacuum (< 10^{-5} mmHg) for 10 h prior to the measurements. Pore size distributions were calculated from the N_2 adsorption isotherms (77 K) using the density functional theory model.

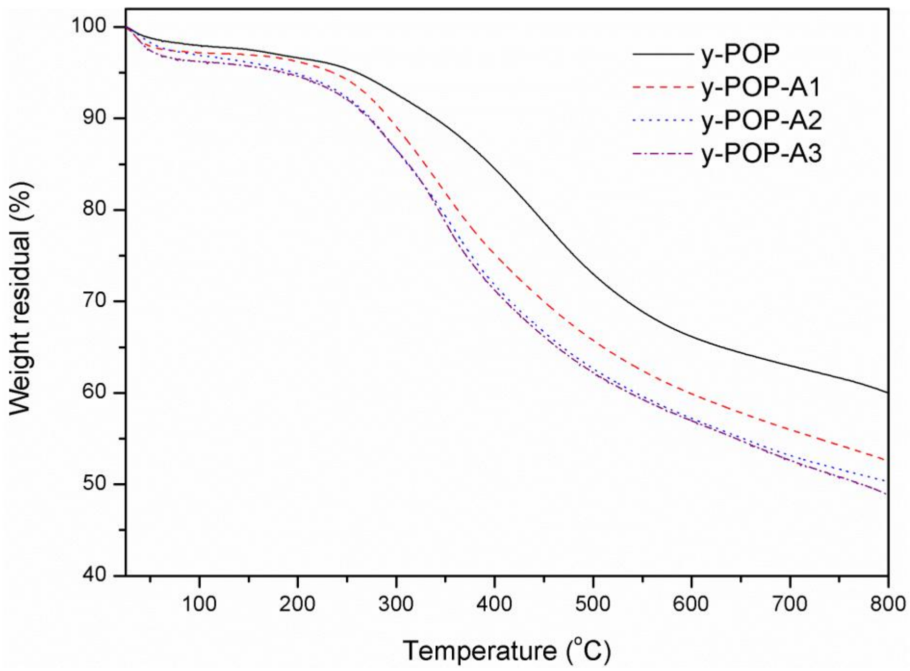

Figure S1. Thermogravimetric analysis curves of y-POP and y-POP-NH_2.

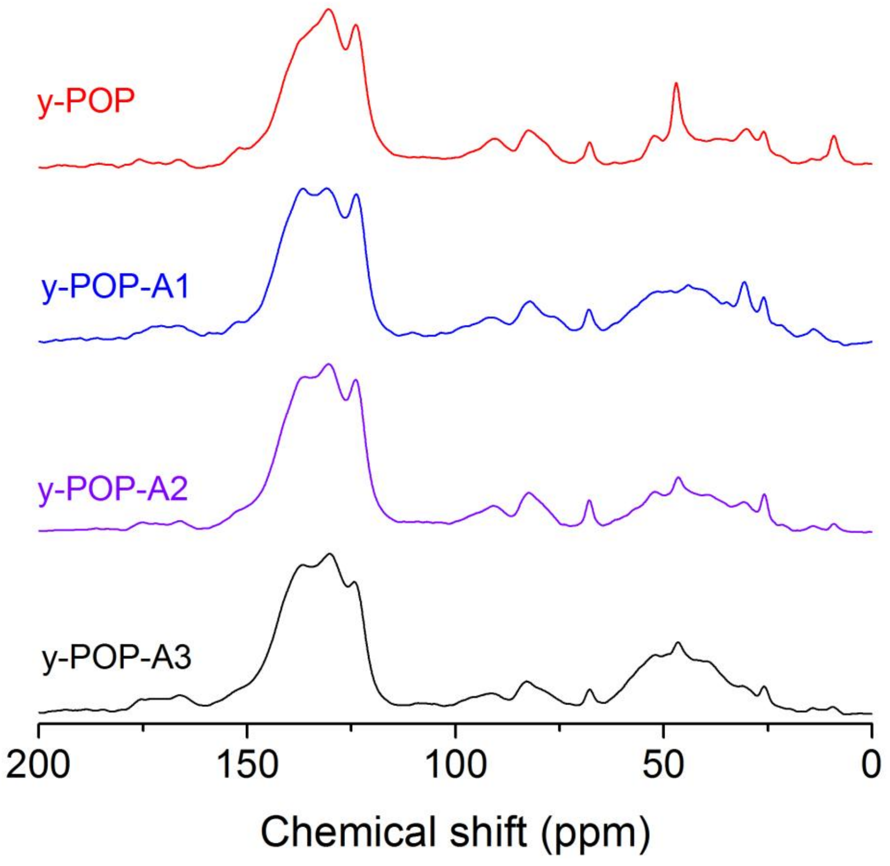

Figure S2. Solid-state ^{13}C NMR spectra of y-POP, y-POP-A1, y-POP-A2, and y-POP-A3.

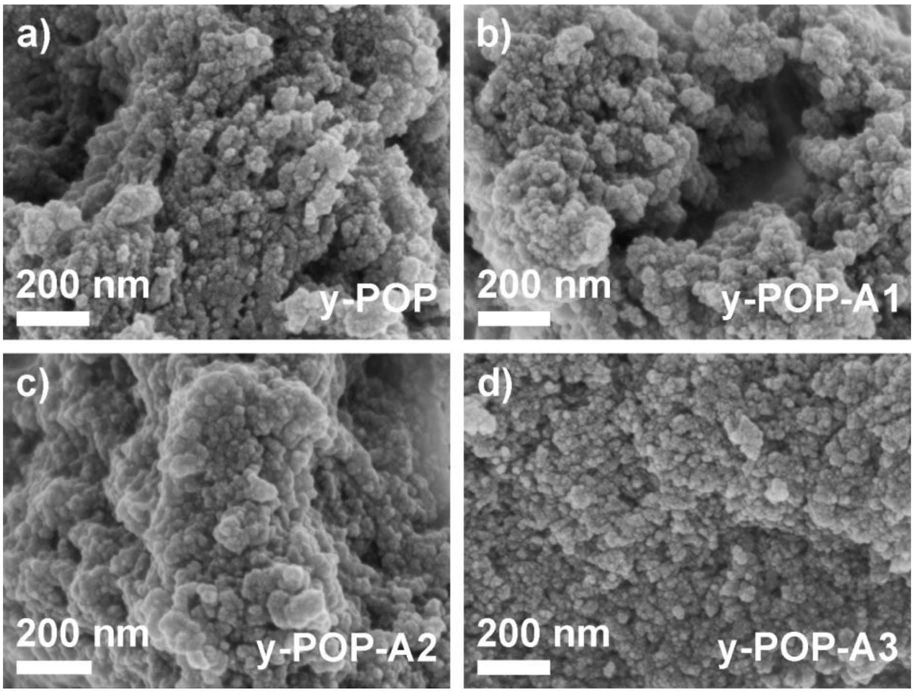

Figure S3. SEM images of y-POP, y-POP-A1, y-POP-A2, and y-POP-A3.

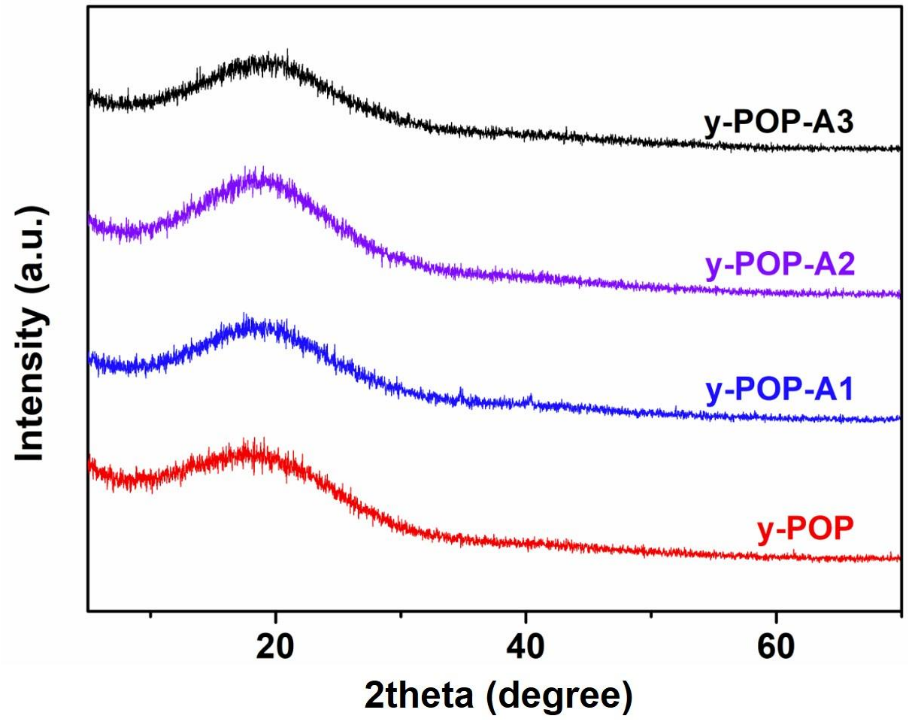

Figure S4. Powder X-ray diffraction patterns of y-POP and y-POP-NH_2 showing the polymers are mainly amorphous.

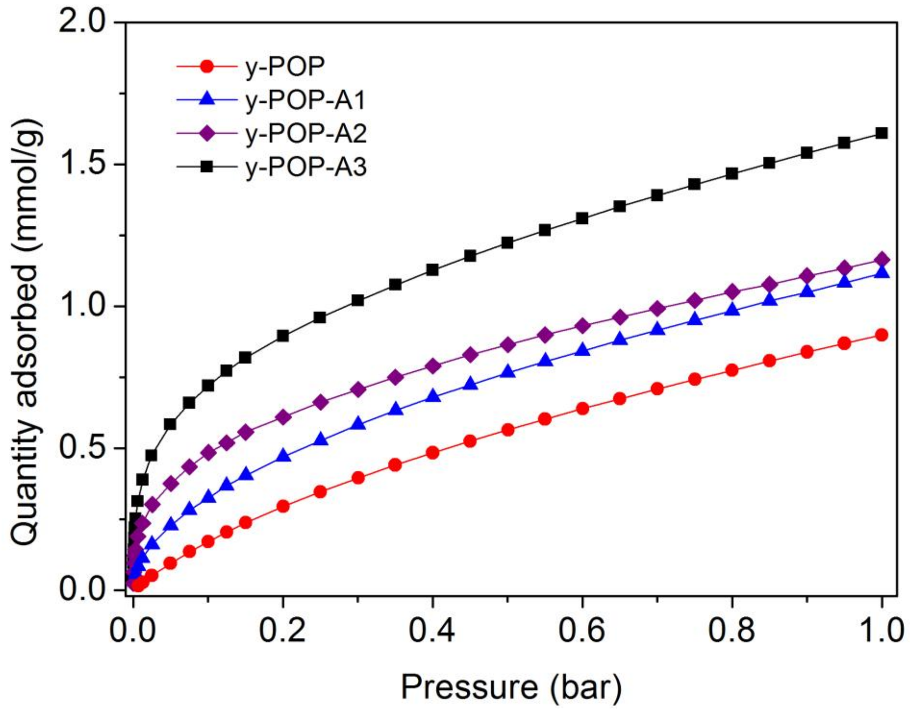

Figure S5. CO_2 adsorption isotherms of y-POP, y-POP-A1, y-POP-A2, and y-POP-A3 recorded at 293 K.

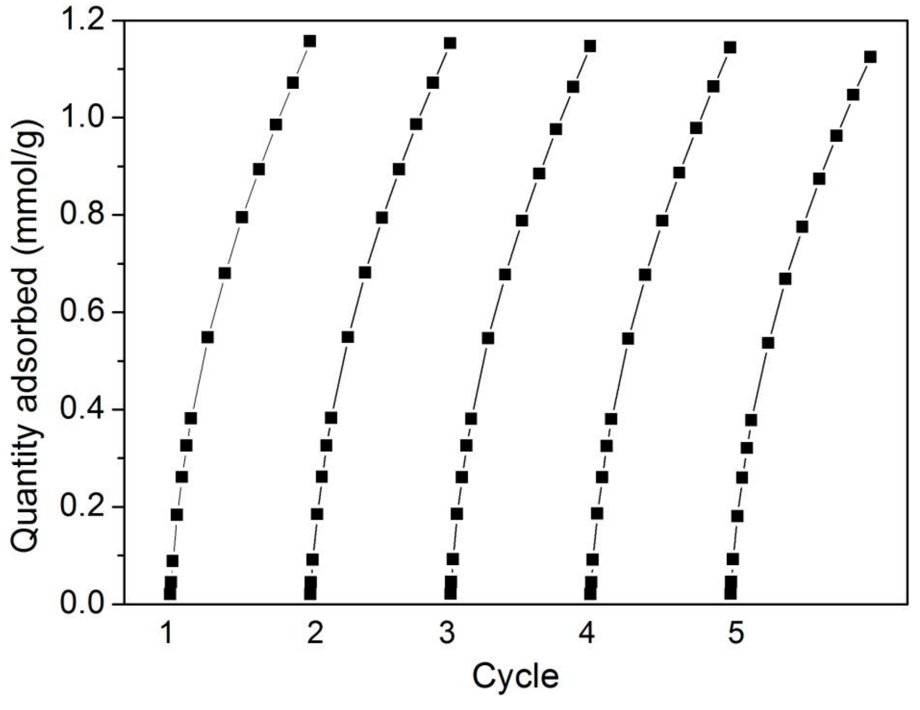

Figure S6. CO_2 adsorption-desorption cycles for y-POP-A1 recorded at 293 K.

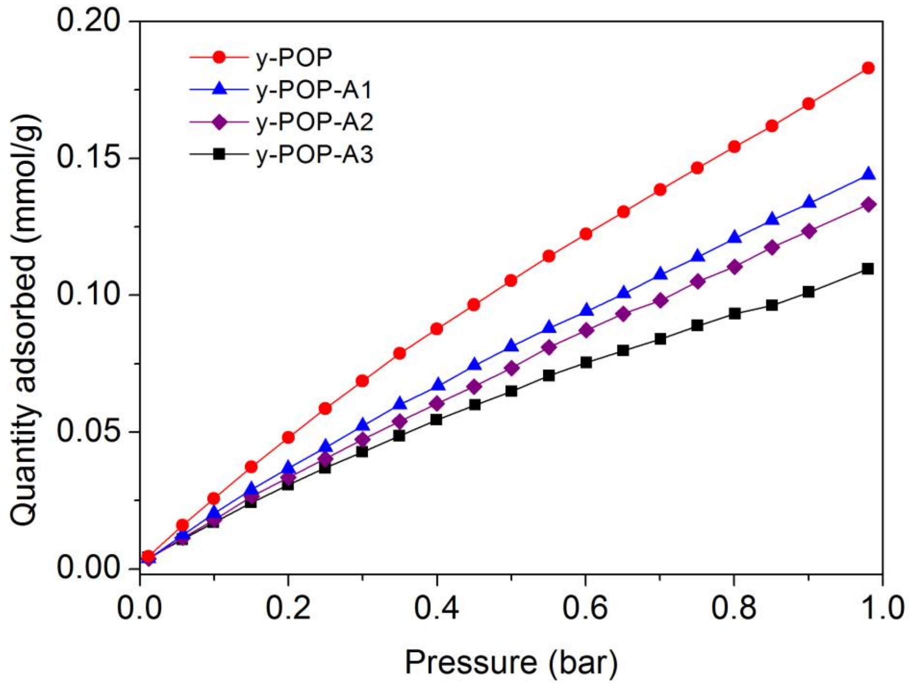

Figure S7. N_2 adsorption isotherms of y-POP, y-POP-A1, y-POP-A2, and y-POP-A3 recorded at 273 K.

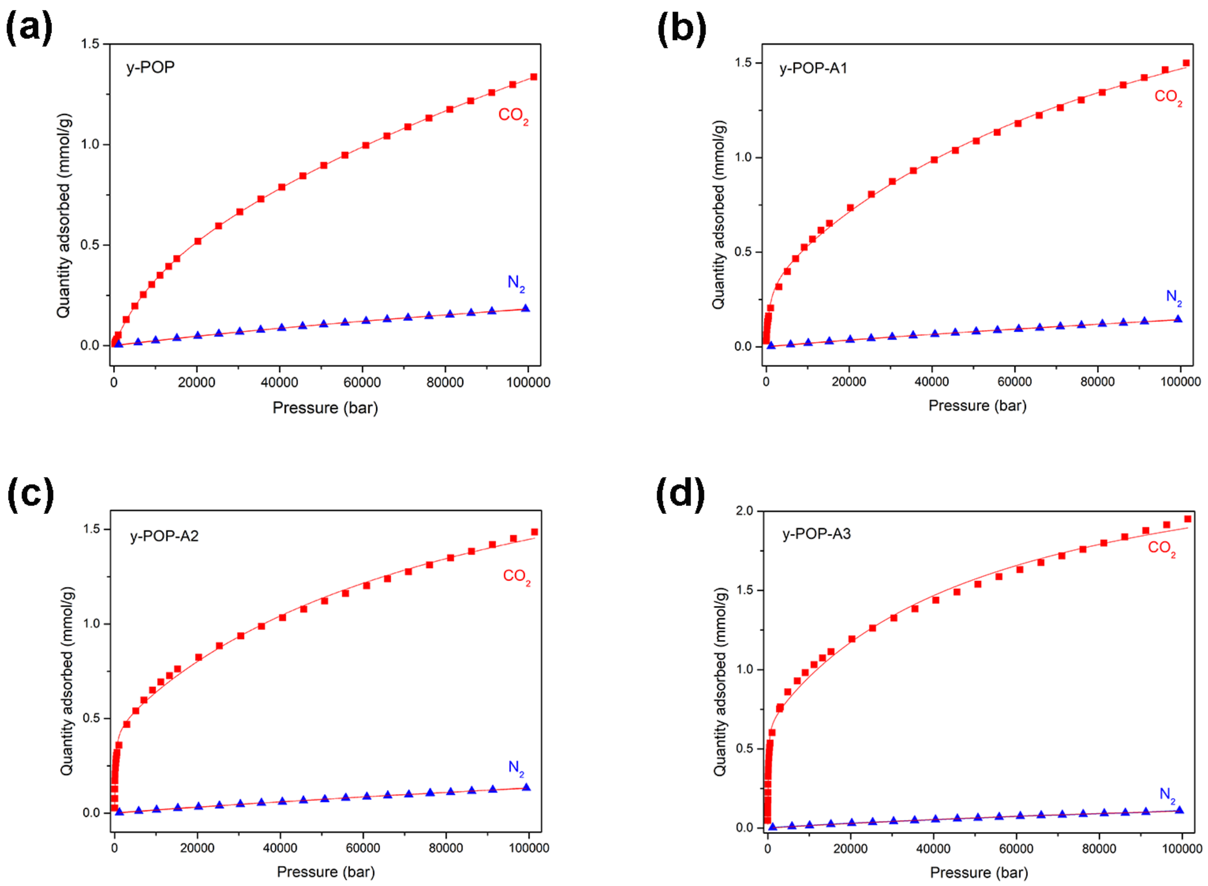

Figure S8. CO_2 (■) and N_2 (▲) adsorption data and of y-POP and y-POP-NH_2 recorded at 273 K. The red solid lines show the fitting results of the data: The CO_2 and N_2 adsorption data was fitted by a dual-site and single-site Langmuir model, respectively. Detail fitting results are given in Table S1. The fitted parameters from the single adsorption data were used to predict the IAST selectivity.

Table S1. Fitting parameters for the CO_2 and N_2 adsorption data recorded at 273 K

Sample	Gas	$q_{sat, A}$ (mmol g^{-1})	b_A (Pa^{-1})	$q_{sat, B}$ (mmol g^{-1})	b_B (Pa^{-1})	Reduced Chi-Sqr	R^2 (COD)	Adj. R-Square
y-POP	CO_2	0.49375 ± 0.01659	7.86E-05 ± 2.812E-6	4.24409 ± 0.20789	2.645E-6 ± 2.088E-7	2.084E-6	0.99999	0.99999
	N_2	0.65634 ± 0.02584	3.81E-6 ± 1.902E-7	-	-	2.88787E -5	0.99948	0.99945
y-POP-A1	CO_2	0.35787 ± 0.01819	0.00135 ± 1.80456E-4	2.26017 ± 0.09986	9.72E-06 ± 9.789E-7	2.77146E -4	0.99895	0.99884
	N_2	0.61075 ± 0.03864	3.047E-6 ± 2.346E-7	-	-	1.69345E -6	0.99911	0.99906
y-POP-A2	CO_2	0.45239 ± 0.1856	0.0051 ± 6.9529E-4	1.82151 ± 0.09871	1.21E-5 ± 1.546E-6	6.19752E -4	0.99736	0.9971
	N_2	0.64277 ± 0.04978	2.59E-06 ± 2.376E-7	-	-	1.5947E- 6	0.99902	0.99896
y-POP-A3	CO_2	0.66511 ± 0.02779	0.01089 ± 0.00179	1.88073 ± 0.09327	1.87E-05 ± 2.683E-6	0.00205	0.99488	0.99444
	N_2	0.31636 ± 0.01347	5.16E-06 ± 2.979E-7	-	-	1.15966E -6	0.99892	0.99886

Note:

Dual-site Langmuir model is used to fit the CO_2 adsorption data

$q = q_{sat, A} * b_A * p/(1+b_A * p) + q_{sat, B} * b_B * p/(1+b_B * p)$, where A and B are distinct adsorption sites

Single-site Langmuir model is used to fit the N_2 adsorption data

$q = q_{sat} * b * p/(1+b * p)$

q adsorption capacity (mmol g^{-1})

q_{sat} saturation adsorption capacity (mmol g^{-1})

p pressure (Pa)

b Langmuir constant (Pa^{-1})

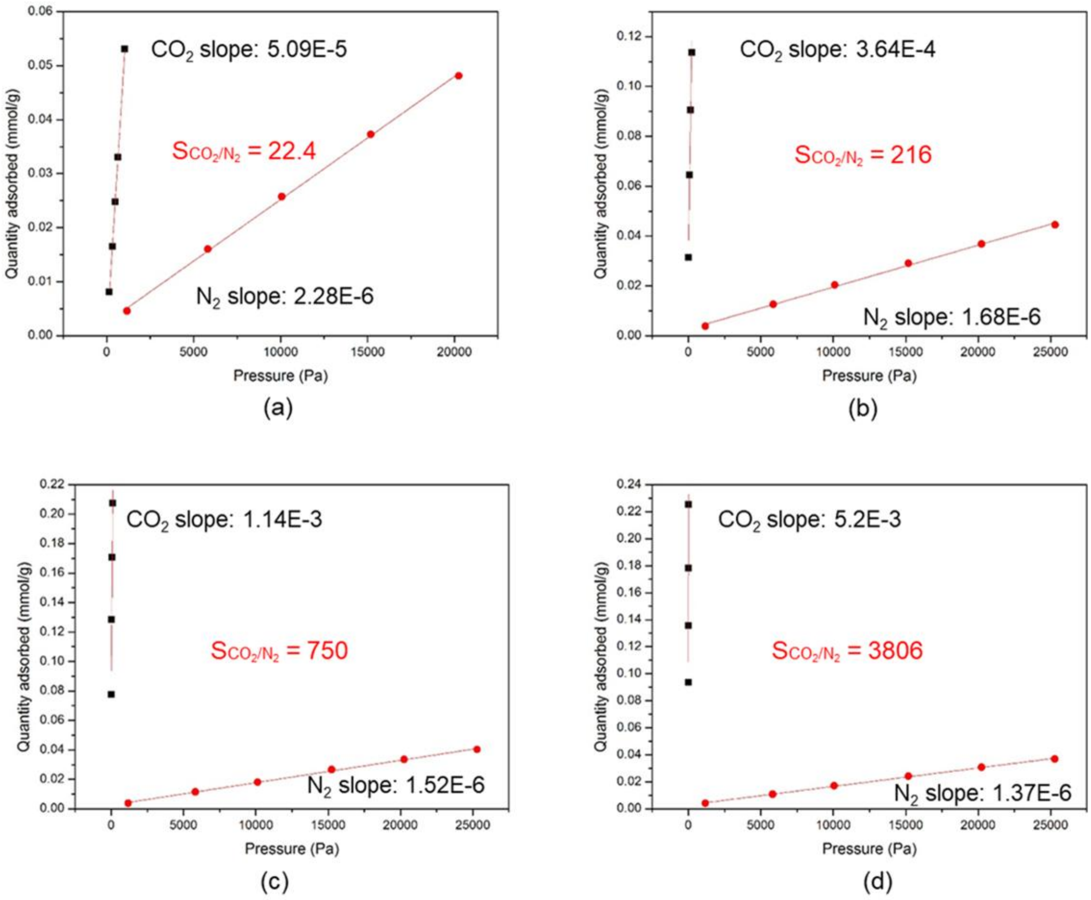

Figure S9. The CO_2 and N_2 adsorption data of (a) y-POP, (b) y-POP-A1, (c) y-POP-A2, and (d) y-POP-A3 at low partial pressures at 273 K and the linearly fitted results. Henry's law CO_2-over-N_2 selectivities were calculated from the initial slopes of the CO_2 and N_2 isotherms.

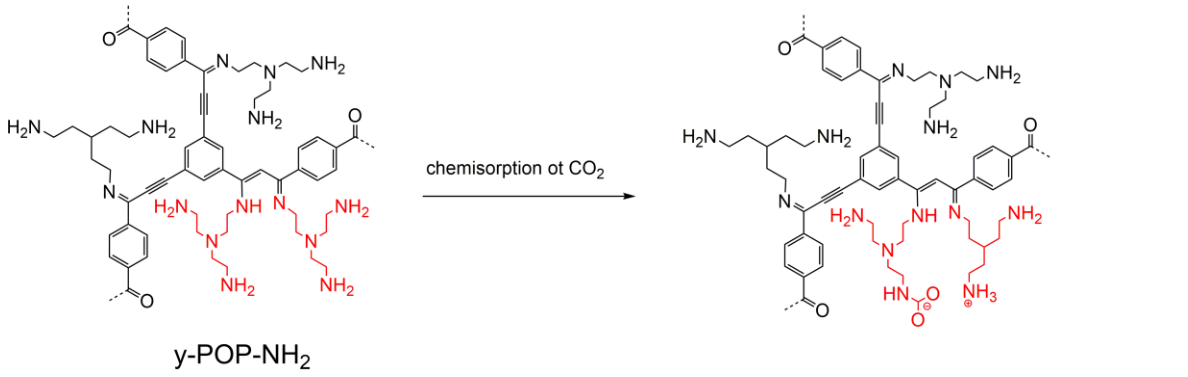

Scheme S1. Possible mechanism of chemisorption of CO_2 on y-POP-NH_2 with high amine loadings.
